# End‐to‐End Deep Learning Framework for Freeform Metasurfaces via Non‐Uniform Rational B‐Splines Meta‐Atoms

**DOI:** 10.1002/nap2.70222

**Published:** 2026-07-29

**Authors:** Kaipeng Liu, Junliang Jia, Kun Li, Yuanjian Wan, Runlin Zhou, Xuyang Chen, Chao Wang, Zhixiang Huang, Jiaxin Li, Gang Gao, Pei Zhang, Longqiu Li

**Affiliations:** ^1^ State Key Laboratory of Robotics and System Harbin Institute of Technology Harbin China; ^2^ Ministry of Education Key Laboratory for Nonequilibrium Synthesis and Modulation of Condensed Matter Xi'an Jiaotong University Xi'an China; ^3^ National Key Laboratory of Science and Technology on Advanced Composites in Special Environments Harbin Institute of Technology Harbin China; ^4^ Chinese Academy of Sciences Shanghai Institute of Optics and Fine Mechanics Shanghai China; ^5^ State Key Laboratory of Opto‐Electronic Information Acquisition and Protection Technology Anhui University Hefei China; ^6^ Department of Electrical and Computer Engineering National University of Singapore Singapore

**Keywords:** deep learning, inverse design, metasurfaces, NURBS

## Abstract

Conventional metasurface designs face fundamental trade‐offs among geometric freedom, fabrication fidelity, and optical functionality. Here, we transcend these limitations through Non‐Uniform Rational B‐Splines (NURBS)‐enabled computational co‐design, establishing a paradigm in which freeform curvature control, deep learning optimization, and nanoscale lithography act synergistically. Unlike topology‐constrained meta‐atoms requiring iterative electromagnetic simulations, our framework deploys a Transformer‐based differentiable surrogate to harness the parametric continuity of NURBS for arbitrary wavefront sculpting. Crucially, we introduce dose curvature proximity compensated lithography, which restores symmetry between design intent and fabricated structure through geometry dependent electron beam modulation. Experimental validation demonstrates achromatic wavelength scale focusing across 405–633 nm, with refined focal plane line profile analysis yielding full‐width‐at‐half‐maximum (FWHM) values of 1.15*λ* ± 0.02*λ* and limited focal shift across the measured wavelength range. The fabricated 200 μm diameter metalens resolves approximately 2.19 μm features in resolution target imaging and enables biological cell image analysis with sensitivity to subcellular refractive index variations (Δ*n* ≈ 0.02). This co‐design platform opens avenues for implantable photonics, microscopy, and quantum manipulation requiring precise freeform architectures.

## Graphical abstract

This work presents a NURBS‐enabled metasurface design framework that combines freeform meta‐atom parameterization with Transformer‐based deep learning. The unified mathematical representation expands geometric degrees of freedom while enabling efficient optical‐response prediction and inverse design. Together with fabrication‐aware optimization, this approach supports achromatic metalens design and provides a scalable route toward high‐performance freeform meta‐optical devices.

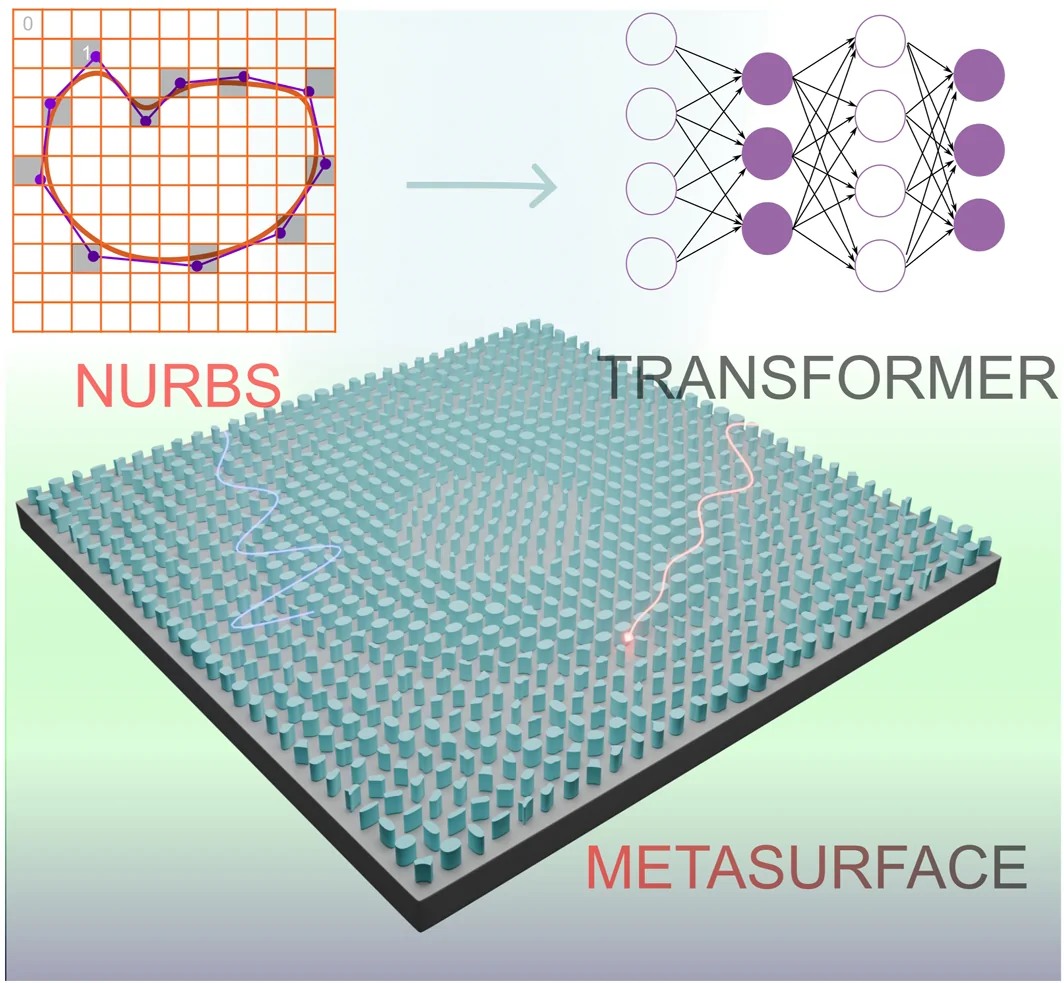

## 1 Introduction

Optical metasurfaces have revolutionized wavefront engineering by enabling arbitrary manipulation of light properties through subwavelength nanostructures, driving innovations in ultracompact imaging, quantum optics, and computational sensing [1, 2, 3, 4, 5, 6, 7, 8]. The core functionality of these devices hinges on the precise design of meta‐atoms that locally control phase, amplitude, and polarization [9, 10]. Despite significant progress, conventional metasurface design strategies remain constrained by a critical trade‐off: geometric simplicity limits optical performance, whereas complex topologies challenge computational optimization and nanoscale fabrication [10, 11].

Current paradigms predominantly employ meta‐atoms with low‐dimensional parametric spaces (e.g., nanopillars and nanofins), which inherently restrict dispersion control and achromatic operation [12, 13, 14]. The conventional approach of constructing composite meta‐atoms by assembling simple geometric primitives (e.g., cuboids) inherently limits design flexibility [15, 16]. This method offers minimal gains in degrees of freedom (DOF) and introduces parametric incompatibility among constituent elements, obstructing unified database implementation. To overcome these limitations, a parametric model enabling unified geometric representation for meta‐atoms is essential. Non‐Uniform Rational B‐Splines (NURBS) provide such a mathematical framework. By adjusting control point parameters, NURBS can represent arbitrary geometric primitives with consistent parameterization. Defining meta‐atom boundaries using NURBS fundamentally expands design DOF while maintaining parametric uniformity. These NURBS‐defined structures constitute a specialized class of freeform meta‐atoms.

Although NURBS freeform geometries offer a theoretical pathway to overcome these limitations, their high‐dimensional optimization imposes prohibitive computational demands [17, 18, 19]. Traditional numerical methods such as finite‐difference time‐domain (FDTD) and finite element method (FEM) simulations require exorbitant resources for devices exceeding > 10^5^ elements, while existing deep‐learning surrogates exhibit limited generalizability beyond low‐dimensional design spaces [20]. Compounding this challenge, standard nanofabrication techniques struggle to maintain structural fidelity across meta‐atoms with varying curvatures and aspect ratios, leading to inconsistent optical responses.

To address these dual bottlenecks in design and manufacturing, we introduce a co‐design framework integrating NURBS based geometric parameterization, Transformer‐accelerated inverse design, and curvature aware nanofabrication [21]. NURBS parameterization unlocks high‐dimensional design freedom through continuous curvature control [22], enabling precise engineering of phase and dispersion relationships. Our Transformer‐based surrogate model bypasses computational bottlenecks by learning electromagnetic responses directly from geometric encodings, significantly accelerating optimization cycles. Crucially, a physics aware fabrication protocol dynamically adapts manufacturing parameters to local feature geometries, ensuring design to device fidelity.

This methodology establishes a new paradigm for computational meta‐optics, where NURBS freeform architectures transcend traditional performance complexity trade‐offs. By synergizing parametric expressiveness, AI‐driven optimization, and manufacturing aware constraints, our work paves the way for high performance metasurfaces in size constrained applications such as endoscopic probes and implantable sensors, where conventional optics prove physically impractical.

## 2 Discussion and Results

### 2.1 Deep Learning‐Enhanced Design of NURBS Meta‐Atoms Based Metasurfaces

Metasurface engineering fundamentally involves balancing phase manipulation and transmission efficiency [1, 23]. The wavefront control in metasurfaces originates from their spatially variant response to incident waves. Distinct meta‐atoms at different positions exhibit heterogeneous optical responses, enabling point by point modulation of both phase and amplitude upon light‐structure interaction. As illustrated in Figure 1a, the metasurfaces modulate incident light by engineering phase retardation and amplitude control through its meta‐atoms. This enables customizable wavefront shaping for beams with diverse wavelengths, polarizations, incident phase profiles, and amplitudes, achieving arbitrarily designed optical wavefronts. In conventional design approaches, the optical response of individual meta‐atoms on a metasurface is obtained through numerical simulations, typically employing FEM or FDTD techniques to solve Maxwell's equations numerically for full field analysis [24, 25]. However, when designing metasurfaces containing up to hundreds of thousands or even millions of meta‐atoms, this approach suffers from three critical limitations [26]: (1) inefficient parallelization of computational resources, (2) mandatory adjoint simulations for sensitivity analysis via the adjoint method [27, 28, 29], and (3) significantly reduced computational efficiency in structural optimization. We therefore introduce deep learning models to supersede conventional numerical algorithms. The innate automatic differentiation capability of deep neural networks enables efficient large scale optimization of metasurface structural parameters. This deep learning framework, typically implemented as an end‐to‐end model (Figure 1b), directly outputs both the optical response and its parameter sensitivities when fed with meta‐atom geometric parameters. Unlike published end‐to‐end approaches for metasurface design [20], we implement Transformer architecture (Figure 1c) as the numerical surrogate model for NURBS based meta‐atoms in our framework. This implementation addresses the higher dimensional parameter space of NURBS meta‐atoms, enabled by their richer geometric features, where Transformer architectures demonstrate superior trainability and compatibility compared to basic autoencoder type neural networks [21]. The input of the Transformer comprises engineered digital encodings that mathematically represent the geometric parameters of NURBS meta‐atoms. As shown in Figure 1d,e, each NURBS meta‐atom constituting the metasurface features a geometric profile bounded by closed NURBS curves. The abundant geometric features and independent curvature degrees of freedom inherent in NURBS enable meta‐atoms to support a significantly richer set of optical eigenmodes, quantitatively expressed as,

(1) $$\Delta N_{\text{modes}}=c\int_{\Omega }\left|\nabla \kappa \left(x,y\right)\right|dV,$$

Here, Δ*N*_modes_ denotes the change in the number of eigenmodes induced by geometric boundary perturbations. The coefficient *c* is constant, and *κ*(*x*, *y*) denotes the curvature of the NURBS meta‐atoms. For the detailed derivation, see Supporting Information S1: Note S1. The formula demonstrates that the number of resonant modes in meta‐atoms is proportional to the number of independent curvature features of the NURBS, providing a theoretical quantification of the mode‐rich characteristics of NURBS based meta‐atoms. Moreover, the multi‐degree‐of‐freedom nature and unified mathematical representation of NURBS meta‐atoms allow a single meta‐atom to be optimized for multiple design objectives, such as phase, amplitude, dispersion, and fabrication constraints, while providing a structured parameter space that can be naturally integrated with deep learning models, including knowledge distillation from existing metasurface databases or surrogate models.

**FIGURE 1:**
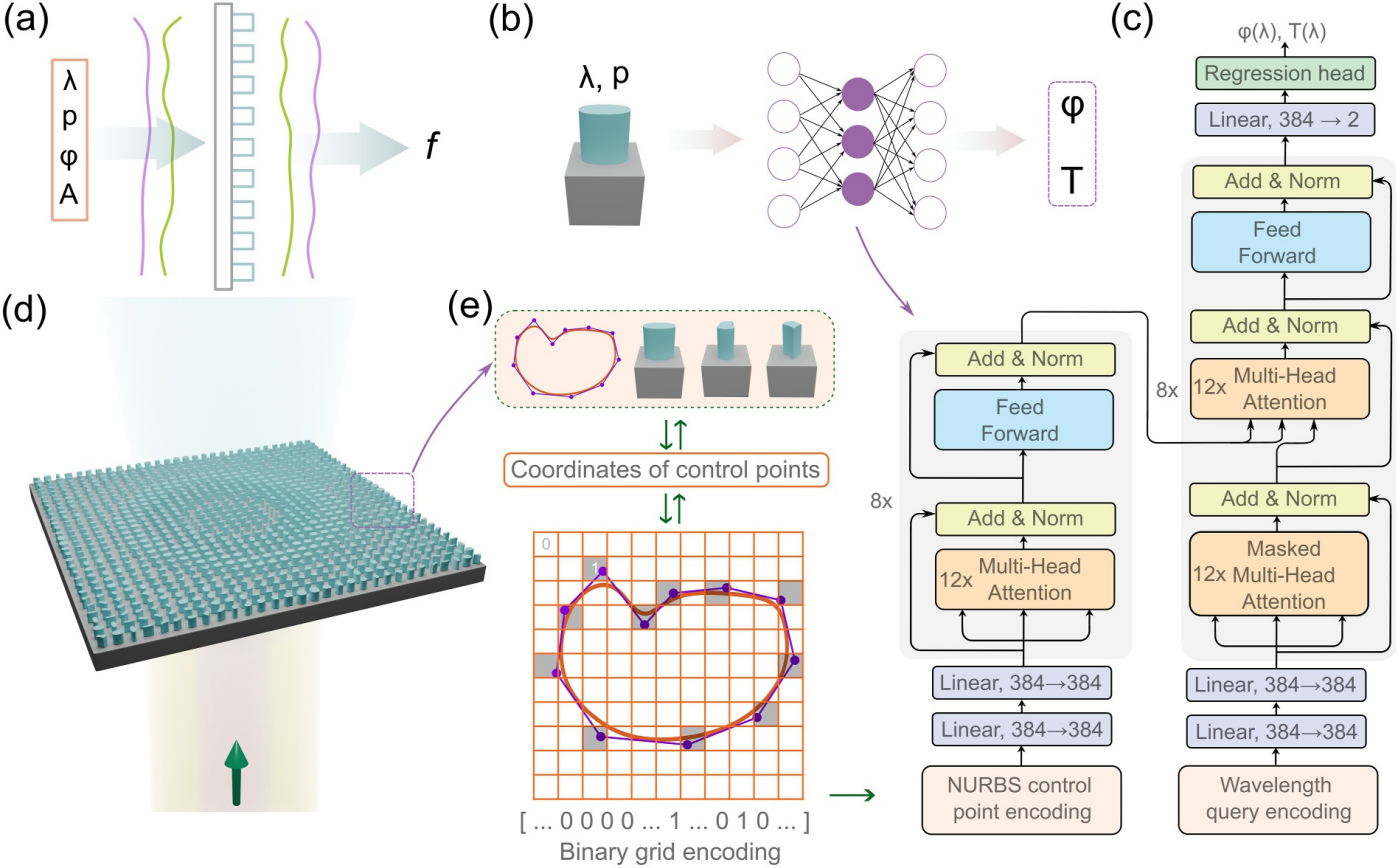
Deep learning assisted design of metasurfaces with NURBS meta‐atoms. (a) Schematic diagram of beam manipulation by a metasurface. (b) The deep learning model maps the geometric parameters of meta‐atoms to their corresponding optical responses. (c) This end‐to‐end Transformer architecture serves as a deep learning surrogate that directly predicts optical responses from input NURBS meta‐atom parameters. (d) Schematic of a metasurface composed of NURBS meta‐atoms. (e) NURBS meta‐atoms. The geometric boundary of the NURBS meta‐atom is enclosed by NURBS curves, where the control points of these curves are distributed on a grid in the Cartesian coordinate system. This facilitates parameter initialization for deep learning training.

For numerical processing efficiency, we discretize the Cartesian coordinate system with grid points, where the control points of NURBS curves are mapped to binary encoded grid indices. The trained Transformer model serves as an FDTD trained surrogate for rapid optical response prediction, enabling accelerated structural optimization of NURBS meta‐atoms while retaining FDTD simulations as the reference data source for training and validation.

Following the NURBS geometric encoding introduced in Figure 1, we constructed an FDTD‐assisted data generation and inverse design workflow, as summarized in Figure 2. In this workflow, each NURBS meta‐atom is simulated as a TiO_2_ unit cell on a SiO_2_ substrate, and the wavelength dependent phase and transmittance are extracted to build the supervised training database. The trained Transformer surrogate then replaces repeated full wave simulations during inverse design, enabling rapid prediction of the optical response and gradient based optimization of the NURBS control point coordinates toward the target phase profile.

**FIGURE 2:**
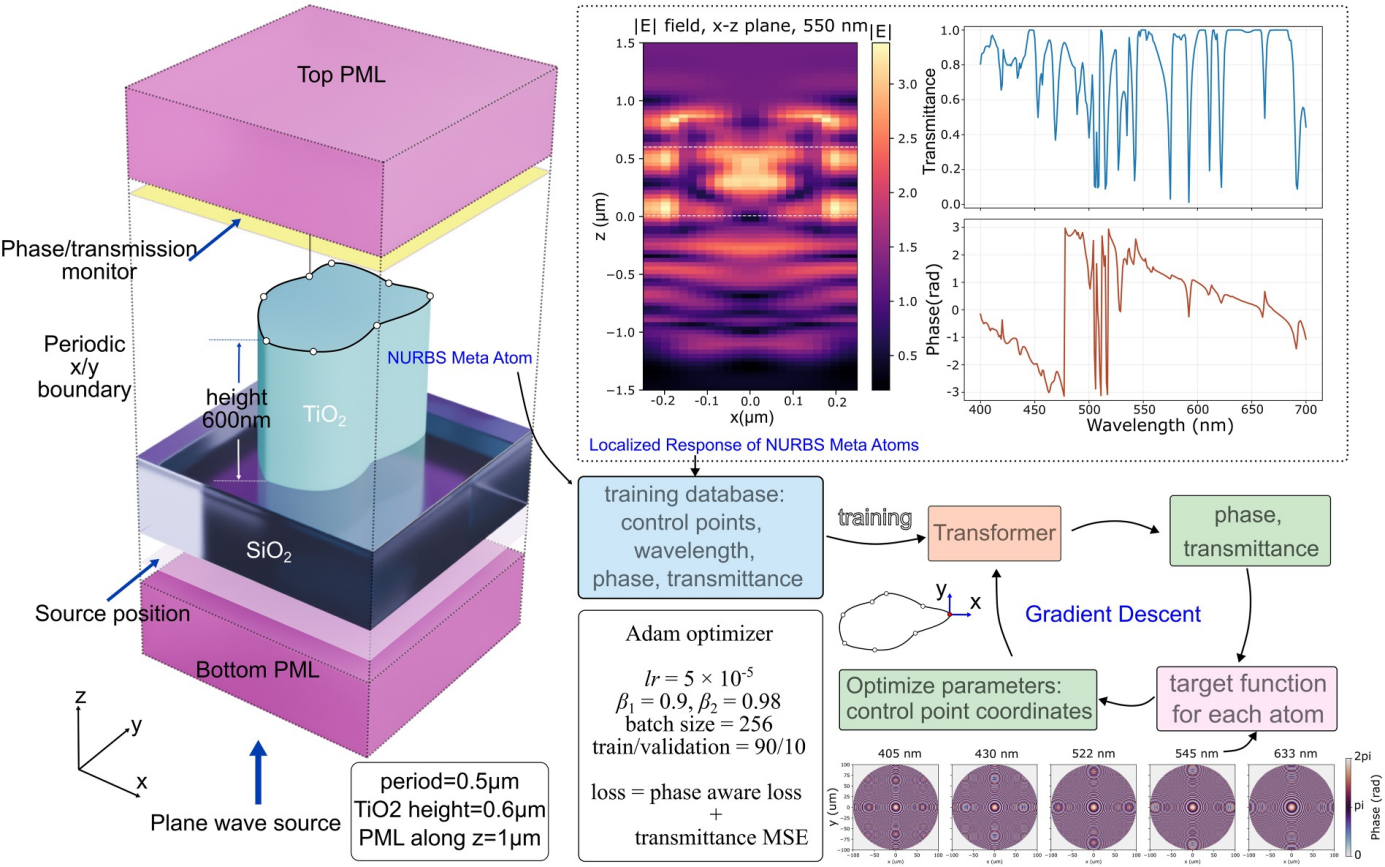
FDTD assisted training and inverse design workflow for NURBS meta‐atoms. The unit‐cell simulation uses a TiO_2_ NURBS meta‐atom on a SiO_2_ substrate with periodic boundary conditions in the lateral directions and PML boundaries along *z*. An incident plane wave is launched from the substrate side, and the phase and transmittance are extracted from a monitor above the meta‐atom. The resulting database, consisting of NURBS control point coordinates, wavelength, phase, and transmittance, is used to train the Transformer surrogate model. The trained surrogate predicts the optical response and enables gradient based optimization of the NURBS control point coordinates toward the target phase function.

The prediction accuracy of different meta‐atom parameterizations is summarized in Table 1, where D‐Flat based nanofin, nanocylinder, and nanoellipse models are used as representative baseline parameterizations [30]. For consistency, all entries are reported using the same optical‐response error definitions, including weighted complex MSE, amplitude MAE, and phase MAE. These design errors were evaluated for the achromatic metalens with a diameter of 200 μm and a focal length of 200 μm at the selected wavelengths (405, 430, 522, 545, and 633 nm), by comparing the target and designed phase profiles as well as the target and designed amplitude distributions. Compared with these conventional geometry classes, the continuous NURBS surrogate achieves substantially lower errors in all evaluated metrics. Specifically, the NURBS‐based model reduces the weighted complex MSE from approximately 1.0 for the D‐Flat baselines to 0.4042, while the phase MAE decreases to 0.5211 rad. These results indicate that the continuous NURBS representation provides a more expressive and learnable design space for modeling freeform meta‐atoms, thereby improving the reliability of the subsequent inverse‐design optimization.

**TABLE 1:** Benchmarking model prediction errors for different meta‐atom parameterizations.

Model	Weighted complex MSE	Amplitude MAE	Phase MAE (rad)
DFlat nanofin	0.9990	0.8357	1.0470
DFlat nanocylinder	1.0541	0.8455	1.1000
DFlat nanoellipse	1.1469	0.9050	1.1715
NURBS surrogate, continuous	0.4042	0.5481	0.5211

*Note:* Lower values indicate better agreement. D‐Flat, a differentiable flat‐optics framework [30], was adopted as the baseline to benchmark conventional nanofin, nanocylinder, and nanoellipse parameterizations. The same error definitions were used for all reported entries.

### 2.2 Processes and Characterization Methods for NURBS Metasurfaces

The improved surrogate accuracy supports efficient inverse design of freeform meta‐atoms, but the resulting geometric diversity also imposes stricter fabrication requirements. NURBS parameterization offers substantially greater geometric flexibility than traditional regular geometries, enabling precise control over light–matter interactions and complex phase profiles within a single metasurface layer. However, this design advantage increases sensitivity to fabrication errors [31, 32, 33], particularly because meta‐atoms with widely varying curvature, area, and neighboring‐pattern environments may respond differently during electron‐beam exposure and pattern transfer [3, 23, 34, 35, 36]. To address this challenge, we developed a variable‐dose electron‐beam lithography process optimized for NURBS meta‐atoms. As demonstrated in Figure 3d, the exposure dose is assigned according to local geometric descriptors, with typical values ranging from 80 to 220 μC/cm^2^ across the metasurface. In process specifications, the EBL dose was correlated with NURBS unit parameters as follows,

(2) $$\text{dose}=k\cdot c\left(x_{i}\right).$$

**FIGURE 3:**
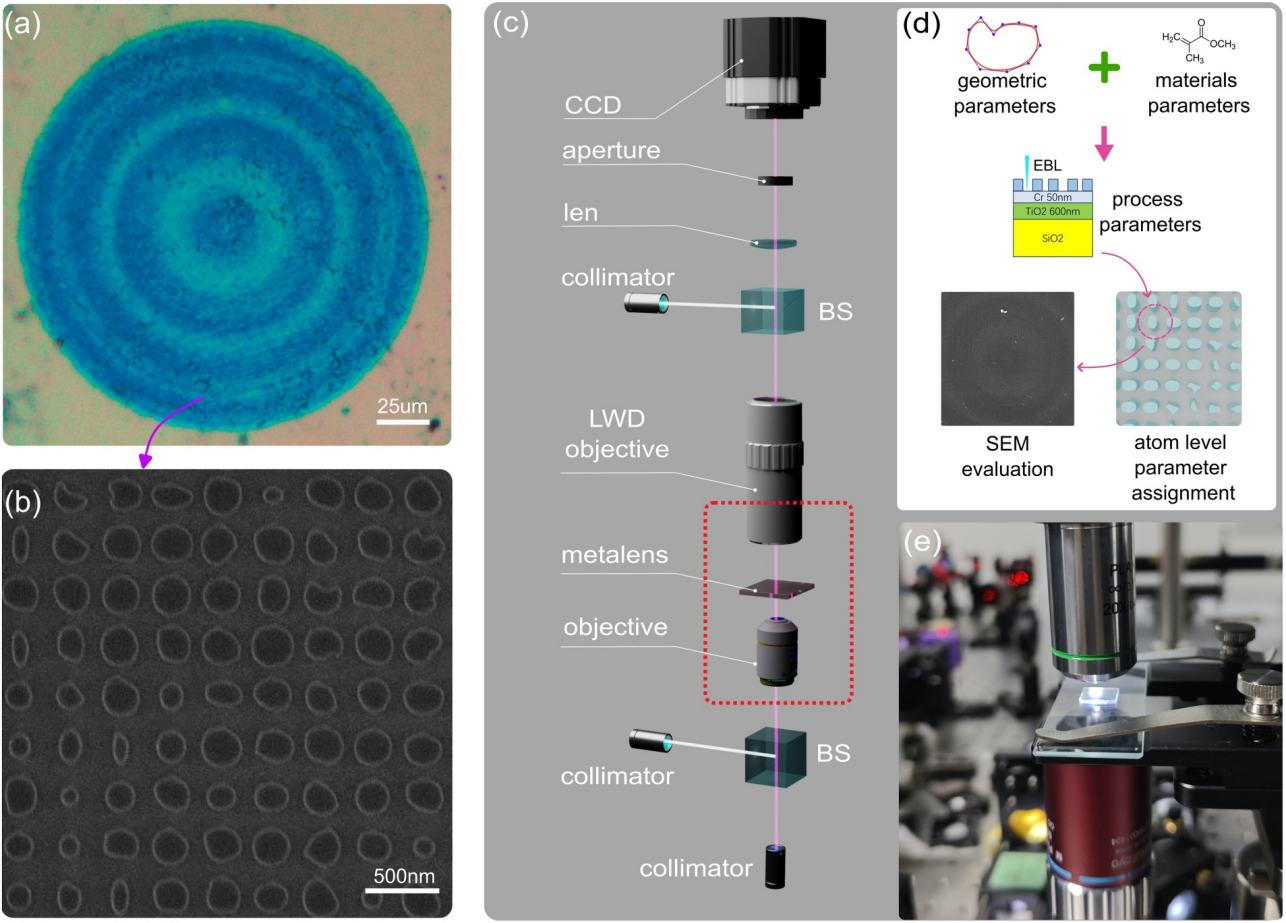
Fabrication and measurement system for NURBS meta‐atom based metasurfaces. (a) Optical microscope image of the metasurface. The scale bar represents 25 μm. (b) Scanning electron microscopy (SEM) image of the fabricated metasurface. The scale bar is 500 nm. (c) Metasurface testing system schematic (support structures and displacement control mechanisms omitted). (d) Fabrication process of NURBS based metasurfaces. Due to significant variations in the geometric features of NURBS meta‐atoms, each unit requires independent assignment of processing parameters (e.g., electron beam exposure dose) during manufacturing to ensure fabrication precision. (e) Zoom‐in of metasurface test system setup.

The EBL exposure dose is defined as a function of the intensity coefficient *k* and the geometric parameters of the NURBS meta‐atom, where *c*(*x*_*i*_) is derived from the control point coordinates *x*_*i*_. Here, *c*(*x*_*i*_) denotes a geometry dependent factor derived from the NURBS control‐point coordinates *x*_*i*_ and is therefore not an independent scalar parameter. The empirical calibration coefficient in the dose‐assignment model can be fitted from process data to relate the NURBS geometric descriptors to the required exposure dose. A more judicious approach is to implement a deep neural network trained on empirical process data [37], the details can be found in Supporting Information S1: Note S2.

In practice, the variable dose EBL strategy was implemented as a geometry dependent exposure assignment for each NURBS meta‐atom. Unlike regular nanofin or nanocylinder arrays, NURBS meta‐atoms contain continuously varying boundaries with different local curvature radii, projected areas, and neighboring‐pattern environments. A uniform exposure dose can therefore lead to nonuniform pattern transfer, particularly near high‐curvature regions or closely spaced features where proximity effects become more pronounced. To compensate for these effects, the exposure dose was adjusted according to the local curvature, meta‐atom area, and proximity environment of each NURBS pattern. This curvature, area, and proximity dependent exposure assignment improves the fidelity between the designed NURBS boundary and the fabricated structure, providing a practical fabrication aware route for transferring freeform meta‐atoms into physical metasurfaces.

The metalens is visually confirmed through optical microscopy imaging in Figure 3a, revealing a uniformly structured 200 μm diameter device. Critical structural details emerge in the SEM micrograph of Figure 3b, which showcases the geometries of individual NURBS defined meta‐atoms. These NURBS atoms exhibit curvature radii ranging from 80 to 400 nm, demonstrating the parametric versatility enabled by NURBS parameterization compared with conventional periodic structures. To characterize the performance of the metalens, we implemented a transmission dispersion test system (Figure 3c) featuring a tunable laser source (405–633 nm range) and a high‐resolution CMOS detector. Figure 3e provides a magnified view of the focal‐region measurement setup, highlighting the experimental configuration used for optical characterization.

Frequency dependent analysis was conducted across five discrete wavelengths (405, 430, 522, 545, and 633 nm) using precision bandpass filters. The numerical simulation results in Figure 4a–e present cross sectional field distributions at these wavelengths, revealing consistent phase profiles with less than *λ*/12 wavefront error. Experimentally, we reconstructed full 3D light fields (Figure 4f–j) through piezoelectric stage translation with 50 nm positional accuracy, acquiring 31 axial planes with an axial step of approximately 6 μm around the designed 200 μm focal distance. This volumetric scanning methodology enabled comprehensive quantification of focal spot evolution, with particular attention to chromatic performance. Both simulated and experimental datasets confirm achromatic operation, showing less than 5% focal shift variation across the tested spectrum.

**FIGURE 4:**
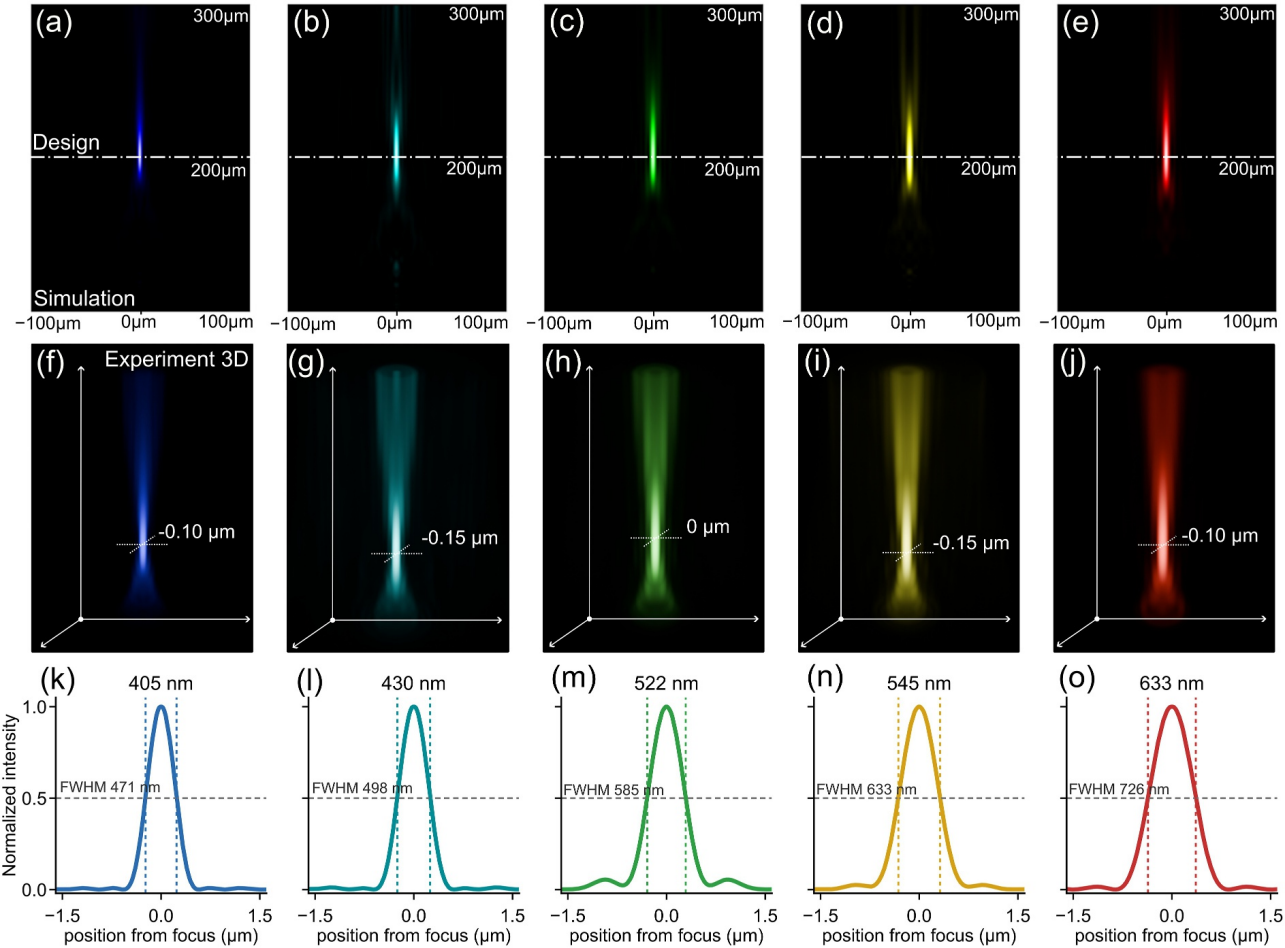
Simulation and experimental results of metasurface optical response. (a–e) Numerical simulation of the optical field distribution after beam transmission through the metasurface. The dashed line indicates the designed focal position. (f–j) Experimental measurement results of the transmitted optical field distribution through the metasurface. (k–o) Measured radial intensity distributions of focal spots at different incident wavelengths.

Quantitative focal spot analysis (Figure 4k–o) further validates the metalens’ performance. The normalized focal plane intensity distributions exhibit FWHM values of 471, 498, 585, 633, and 726 nm at 405, 430, 522, 545, and 633 nm, respectively, corresponding to wavelength‐normalized focal widths of 1.12*λ*–1.16*λ* with an average value of 1.15*λ* ± 0.02*λ* across the measured wavelengths. This spatial confinement originates from the ability of NURBS meta‐atoms to precisely engineer wavefront curvature while minimizing spherical aberrations. The Gaussian‐like intensity profiles observed at each wavelength confirm the absence of significant side lobes or structured background, indicating high‐quality phase control throughout the operational bandwidth. Collectively, these results demonstrate how NURBS parameterization enables simultaneous optimization of geometric diversity, achromatic performance, and diffraction‐limited focusing, capabilities that traditionally require complex multi‐element refractive systems.

### 2.3 Applications for NURBS Metasurfaces in Microscopic Imaging

The development of an achromatic metalens based on NURBS meta‐atoms provides a compact route toward miniaturized imaging systems. As demonstrated in Figure 5a, the NURBS metalens was integrated into a transmission imaging configuration for imaging resolution targets and biological cells. Figure 5b,c compare the USAF‐1951 resolution target images acquired without and with the metalens, respectively, showing that the metalens can resolve features down to approximately 2.19 μm using a single 200 μm diameter optical element.

**FIGURE 5:**
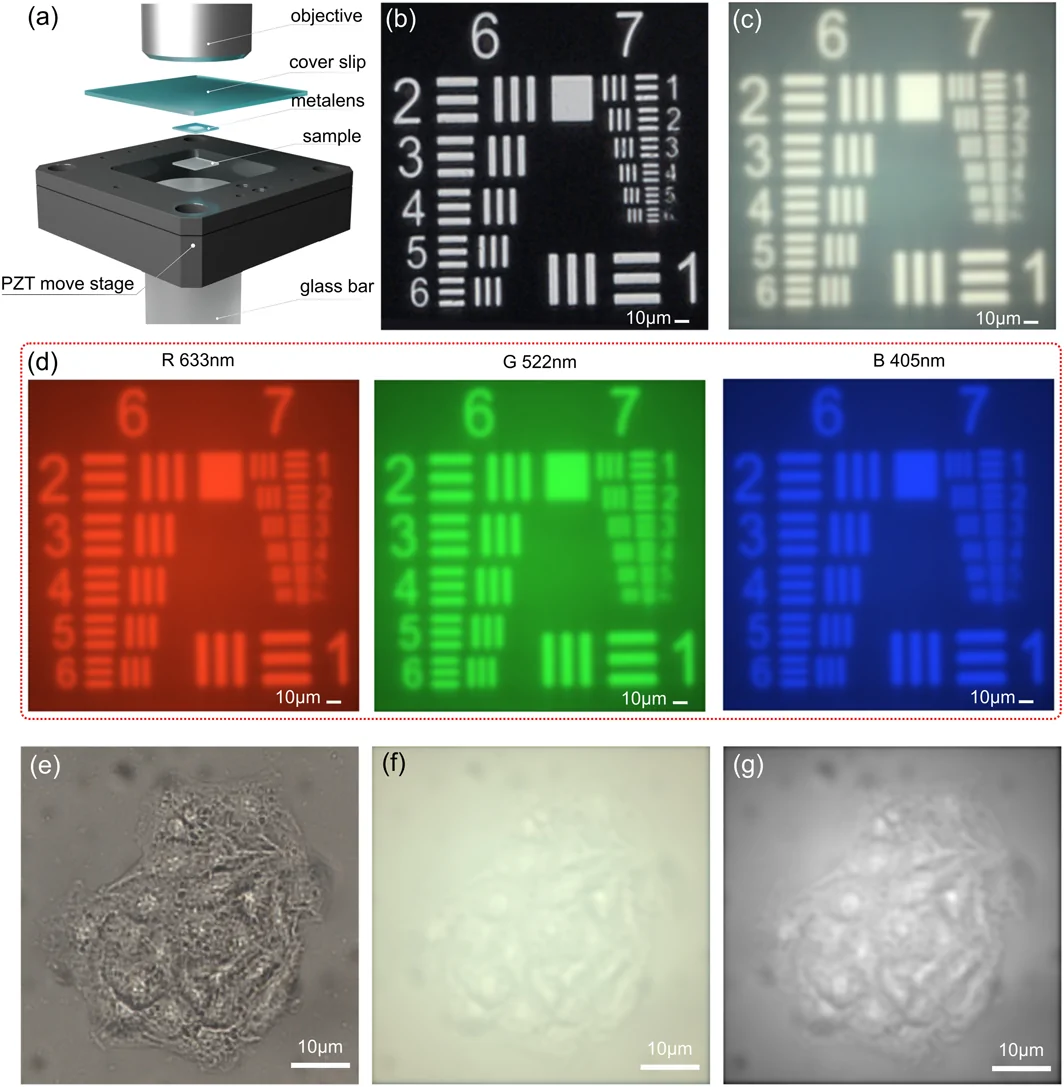
Applications of metasurfaces in microscopic imaging. (a) Imaging test system incorporating a piezoelectric translation stage. (b) Image of the test resolution target without the metalens. (c) Imaging results of the resolution target after the metalens. (d) Metalens imaging results under laser illumination at different wavelengths. (e) Image of Panc02 murine pancreatic cancer cells without metalens. (f) Imaging of Panc02 murine pancreatic cancer cells after the metalens. (g) Grayscale image of Panc02 murine pancreatic cancer cells imaged through the metalens.

When imaging the USAF‐1951 resolution target, the metalens (Figure 5c) achieves a resolution comparable to that obtained without the metalens in the reference microscope image (Figure 5b), resolving features down to approximately 2.19 μm. Broadband imaging performance is further demonstrated under 633, 522, and 405 nm illumination (Figure 5d), where the main features of the resolution target remain recognizable across the visible wavelengths. This wavelength tolerant imaging behavior is consistent with the achromatic focusing performance characterized in Figure 4 and arises from the ability of NURBS meta‐atoms to jointly engineer phase and dispersion through freeform boundary control.

Biological validation further illustrates the practical utility of the NURBS metalens. For Panc02 murine pancreatic adenocarcinoma cells (ATCC CRL‐2552), Figure 5e shows the reference microscope image, Figure 5f shows the corresponding metalens acquired image, and Figure 5g presents the grayscale processed metalens image used for image analysis. The planar form factor and reduced optical complexity of the NURBS metalens platform make it promising for compact imaging systems, including endoscopic probes, wearable diagnostics, and integrated biomedical imaging devices.

### 2.4 Comparative Evaluation of Design Freedom and Fabrication Feasibility

To systematically evaluate the performance of our NURB based approach against existing metasurface design methodologies, we conducted a comparative analysis across two critical dimensions: design degrees of freedom (DOF) and fabrication feasibility (FF). Here, the design DOF is defined as the number of independently adjustable geometric parameters within one meta‐atom or unit cell. For example, a cylindrical meta‐atom has one DOF corresponding to its radius, while a rectangular nanofin has two DOF corresponding to its length and width. For composite units, the total DOF is the sum of the geometric parameters of all constituent primitives. For a NURBS meta‐atom, the adjustable parameters are the control‐point coordinates; therefore, a NURBS boundary with N control points has 2N geometric DOF from the *x* and *y* coordinates of the control points. The FF is an estimated relative index rather than a directly measured fabrication yield. It is assigned according to the expected lithographic difficulty of the pattern, mainly considering the minimum boundary curvature radius, required patterning resolution, boundary smoothness, sharp corners, and proximity‐effect sensitivity. Structures with smooth boundaries, large curvature radii, and low spatial‐frequency features are assigned higher FF, whereas patterns with random boundaries, very small curvature radii, sharp corners, or high resolution nanoscale features are assigned lower FF.

As illustrated in Figure 6, the metasurface design landscape can be categorized into four representative approaches. Traditional geometric methods [1, 6, 9, 38, 39, 40], which use simple parametric shapes such as nanopillars and nanofins, offer limited design flexibility (DOF: 1–3) but maintain relatively high fabrication feasibility (60–75%) because of their compatibility with standard nanofabrication processes. Composite‐structure approaches [41] extend the design space to 5–8 DOF with improved fabrication feasibility (75–80%) by combining multiple geometric primitives. Machine‐learning‐based methods [42, 43, 44, 45], including generative adversarial networks and diffusion probabilistic models, can increase design freedom to 50–100 DOF while maintaining excellent fabrication feasibility (80–90%), although they require extensive training datasets and computational resources. In contrast, topology optimization approaches [46, 47, 48], despite offering the highest DOF (≥ 100), often suffer from compromised fabrication feasibility (30–60%) because irregular freeform geometries with nanoscale features remain challenging for current lithography processes.

**FIGURE 6:**
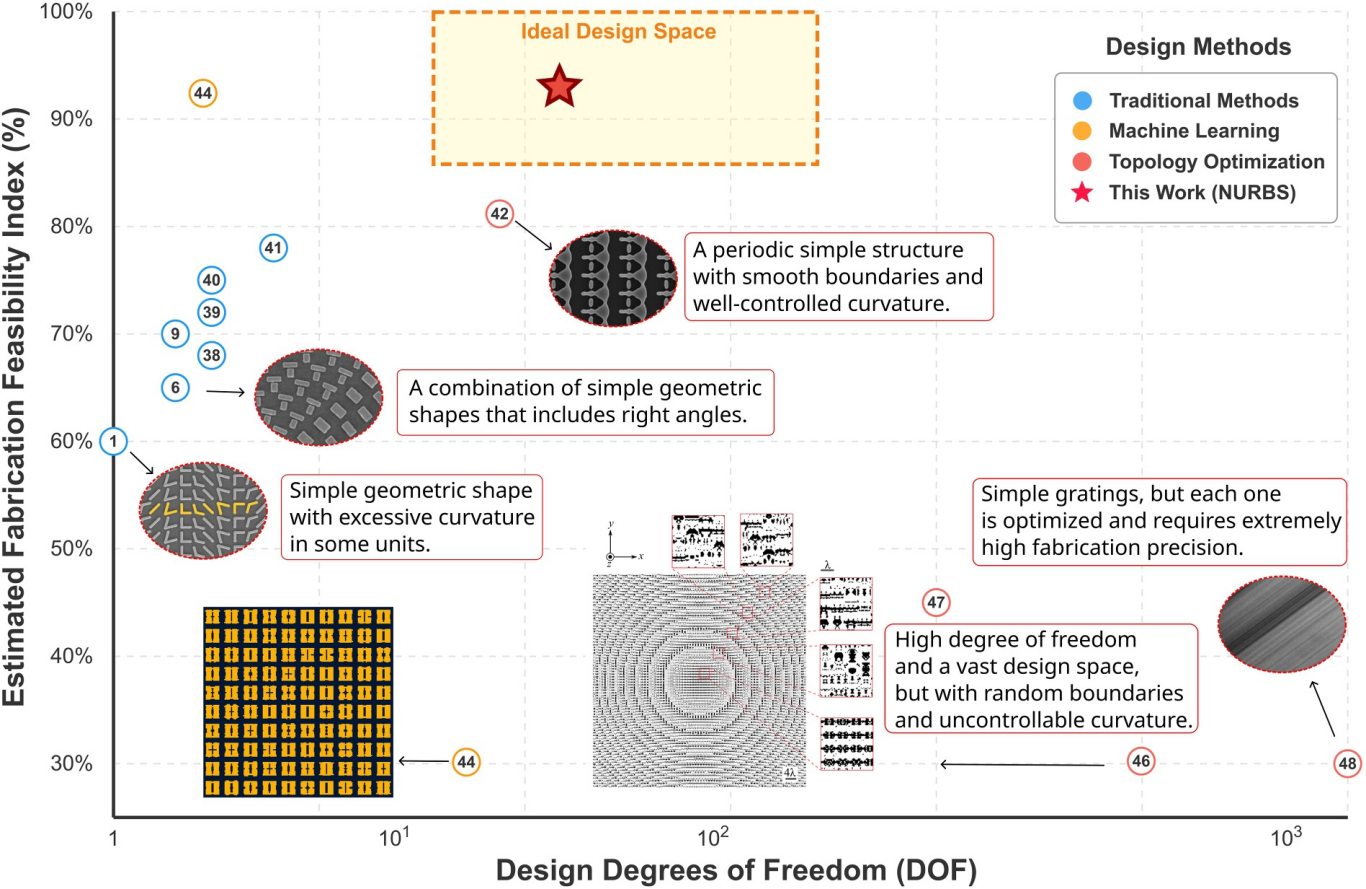
Metasurface design methods: DOF versus estimated fabrication feasibility. We conducted a comparison of design methods using the dimensions of manufacturing difficulty and design freedom. The numbers in the circles indicate the corresponding references. DOF values were assigned according to the number of independently adjustable geometric variables in each design method. The vertical axis represents an Estimated Fabrication Feasibility Index, which is a relative estimate based on feature size, curvature radius, boundary smoothness, and expected lithographic complexity rather than a directly measured fabrication yield.

Our NURBS meta‐atom approach occupies a unique position in this design space, achieving about 50 DOF, substantially higher than traditional geometric methods while remaining computationally tractable, with an estimated fabrication‐feasibility index of 95%. This high manufacturability estimate stems from the inherent smoothness and continuity of NURBS parameterization, which naturally produces fabrication‐friendly geometries compatible with variable‐dose electron‐beam lithography. For NURBS‐based meta‐atoms, each optimized degree of freedom aligns precisely with the structural sensitivity (Supporting Information S1: Note S3). Even without relying on deep‐learning surrogate models to predict unit performance, one can intuitively evaluate whether the current fabrication outcome meets the required optical resonance mode. The combination of moderate‐to‐high design flexibility with strong estimated fabrication feasibility positions our method in the ‘ideal design space’ indicated by the yellow region in Figure 6, thereby bridging the gap between design expressiveness and manufacturing practicality that has constrained previous metasurface optimization frameworks.

## 3 Conclusion

This work establishes a transformative framework for high performance metasurface engineering through the integration of NURBS based meta‐atom design with deep learning enhanced optimization and precision nanofabrication. By harnessing the geometric versatility of NURBS parameterization, we have demonstrated achromatic metalens that overcome fundamental limitations in conventional diffractive optics while achieving unprecedented miniaturization. The Transformer based surrogate model enables efficient optimization of complex freeform structures, reducing design cycles compared to traditional methods. Crucially, our variable dose EBL process successfully translates these intricate designs into functional devices, with measured wavefront errors below *λ*/15 across a 228 nm bandwidth.

Experimental validation confirms wavelength‐scale achromatic focusing across the visible spectrum. The measured focal‐plane intensity profiles show FWHM values of 471, 498, 585, 633, and 726 nm at wavelengths of 405, 430, 522, 545, and 633 nm, respectively, corresponding to normalized focal widths of 1.12*λ*–1.16*λ*. The measured focal positions remain close to the designed 200 μm focal plane across the tested wavelengths, indicating limited chromatic focal dispersion. In imaging applications, the 200 μm diameter metalens resolves features down to approximately 2.19 μm in a USAF‐1951 resolution target and enables grayscale‐based image analysis of cellular morphology, while maintaining a compact planar form factor. These capabilities stem from the synergistic combination of NURBS freeform curvature control, computational fabrication co‐design, and precision nano‐positioning.

The demonstrated technology opens avenues for next‐generation portable endoscopes, wearable diagnostics, and implantable biosensors where size and weight constraints preclude conventional optics. Future work will focus on scaling fabrication throughput and integrating dynamic reconfigurability. By fundamentally redefining the trade‐offs between optical performance, system compactness, and manufacturability, this research provides a foundation for ubiquitous computational optics in biomedical imaging and beyond.

## 4 Methods

### 4.1 Computational Framework

The training dataset was generated through rigorous finite‐difference time‐domain (FDTD) simulations using MEEP. For each unit‐cell simulation, the NURBS TiO_2_ meta‐atom was placed on a SiO_2_ substrate with a lattice period of 0.5 μm and a TiO_2_ height of 0.6 μm. Periodic boundary conditions were applied along the *x* and *y* directions, and perfectly matched layers (PMLs) with a thickness of 1 μm were used along the *z* direction. A normally incident plane wave was launched from the substrate side, and the transmitted field was collected by a monitor positioned above the meta‐atom to extract the wavelength‐dependent phase and transmittance. The simulations were performed over the 400–700 nm wavelength range with a mesh size of 10 nm. The wavelength‐dependent optical constants of TiO_2_ and SiO_2_ were taken from the RefractiveIndex.INFO optical‐constants database, and were implemented in MEEP as dispersive material parameters over the 400–700 nm wavelength range. We simulated 500,000 unique NURBS meta‐atom geometries spanning curvature radii of 50–300 nm and aspect ratios of 0.2–5.0. Each simulation solved Maxwell’s equations on a 10 nm mesh grid across 400–700 nm spectrum, extracting full field solutions with < 0.5% residual error. Geometric parameters were encoded as feature vectors containing control point coordinates, weights, and knot vectors. A Transformer‐based surrogate model was implemented in PyTorch 2.0 using 12 attention heads and 8 encoder layers as shown in Figure 1c. The model was trained on NVIDIA A100 GPUs with 500,000 samples (90% training, 10% validation), optimized via Adam (*lr* = 5 × 10^−5^, *β*_1_ = 0.9, *β*_2_ = 0.98). Automatic differentiation enabled end‐to‐end optimization of the dual‐output architecture predicting both complex optical response (amplitude/phase) and parametric gradients. Training convergence occurred at 10,000 epochs with validation loss plateauing at mean absolute error 0.0187.

### 4.2 Nanofabrication Process

The thin film substrate used for fabricating the metalens was SiO_2_, on which a 600 nm thick TiO_2_ layer was deposited via magnetron sputtering and atomic layer deposition (ALD) methods. The first 500 nm of TiO_2_ was deposited by magnetron sputtering, followed by an additional 100 nm grown via ALD. Finally, a 50 nm thick Cr layer was grown by magnetron sputtering. Metalens fabrication was carried out using a commercial TESCAN EBL system operated at 50 kV with a beam current of 30 pA on samples coated with 220 nm PMMA (2AR‐P 679.04) resist. The layout‐dependent exposure‐dose map was generated using a KLayout based dose correction plugin developed in collaboration with TESCAN, following the geometry‐dependent dose assignment described by Equation (2). The plugin assigns local exposure doses based on the NURBS meta‐atom boundary curvature, projected pattern area, and neighboring‐pattern proximity to compensate for geometry‐dependent exposure and proximity effects during pattern writing. The corrected exposure dose varied from 80 to 220 μC/cm^2^ across the metasurface. It should be noted that the present fabrication‐aware correction mainly addresses lateral pattern‐transfer errors arising from EBL exposure and development. Etching‐related deviations, including TiO_2_ etch bias, sidewall angle variation, sidewall roughness, and height nonuniformity, can also influence the final optical response but were not explicitly included in the present inverse design optimization. Incorporating calibrated etch‐bias and sidewall profile models into the NURBS design workflow will be pursued in future work.

Post exposure, samples underwent development in MIBK:IPA (1:3) for 60 s and in MIBK:IPA (1:4) for 40 s. The patterned sample was first etched in Cl_2_‐based inductively coupled plasma reactive ion etching (ICP‐RIE 20 sccm Cl_2_, 5 mTorr, 200 W RF power) to remove the 50 nm Cr layer, followed by 600 nm TiO_2_ etching using C_4_F_8_‐based ICP‐RIE (25 sccm, 5 mTorr, 300 W RF power) with an Oxford Instruments system. The post‐etch cleaning procedure involved ultrasonic agitation in organic solvents: acetone (≥ 99.5%), IPA (≥ 99.7%), and anhydrous ethanol (≥ 99.9%), each for 5 minutes followed by N_2_ blow drying. Final structures were cleaned via O_2_ plasma bashing and characterized through scanning electron microscope (SEM).

### 4.3 Optical Characterization

A custom transmission mode testbed with piezoelectric nano positioning quantified metalens performance. Tunable laser illumination passed through linear polarizers and quarter‐wave plates before sample interaction. Transmitted fields were imaged using a 100 × NA 0.55 objective onto a CMOS camera. During the measurement process, a linear piezoelectric stage with 50 nm precision was utilized. The preparation method of mouse pancreatic cancer cells can be found in Supporting Information S1: Note S5.

## Author Contributions

**Kaipeng Liu:** writing – review and editing, methodology, software, conceptualization, investigation, writing – original draft, formal analysis, data curation, project administration. **Junliang Jia:** conceptualization, investigation, writing – original draft, writing – review and editing, methodology, data curation. **Kun Li:** data curation, methodology, writing – review and editing, conceptualization, investigation, writing – original draft. **Yuanjian Wan:** conceptualization, investigation, writing – review and editing, methodology. **Runlin Zhou:** conceptualization, investigation, writing – review and editing, methodology. **Xuyang Chen:** conceptualization, investigation. **Chao Wang:** conceptualization, investigation, writing – review and editing. **Zhixiang Huang:** writing – review and editing, conceptualization, methodology. **Jiaxin Li:** conceptualization, investigation, writing – review and editing, methodology, formal analysis, data curation, supervision, resources. **Gang Gao:** conceptualization, investigation, methodology, writing – review and editing, project administration, funding acquisition, supervision. **Pei Zhang:** supervision, funding acquisition, investigation, conceptualization, writing – review and editing, visualization, validation, methodology, project administration. **Longqiu Li:** supervision, resources, project administration, writing – review and editing, funding acquisition, conceptualization, investigation, validation, methodology, visualization.

## Supporting information


Supporting Information S1

## Data Availability

The data that support the findings of this study are available from the corresponding author upon reasonable request. And the code used in this work can be found on the website https://github.com/kaipengliuHIT/nurbs‐meta‐atoms.
