# Supplementary material for: End‐to‐End Deep Learning Framework for Freeform Metasurfaces via Non‐Uniform Rational B‐Splines Meta‐Atoms
Source: Nanophotonics. 2026 Jul 29;15(15):e70222. doi: 10.1002/nap2.70222 (PMC13642555; doi:10.1002/nap2.70222)
Supplement: Supplementary file 1 [file NAP2-15-e70222-s001.docx]

Supporting Information

**End-to-End Deep Learning Framework for Freeform Metasurfaces via Non-Uniform Rational B-Splines Meta-atoms**

Kaipeng Liu^1,†^, Junliang Jia^2,†^, Kun Li^3,†^, Yuanjian Wan^4^, Runlin Zhou^2^, Xuyang Chen^1^, Chao Wang^5^, Zhixiang Huang^5^, Jiaxin Li^6,*^, Gang Gao^3,*^, Pei Zhang^2,*^, Longqiu Li^1,*^

^1^Harbin Institute of Technology, State Key Laboratory of Robotics and System, Harbin, China, 150001

^2^Xi’an Jiaotong University, Ministry of Education Key Laboratory for Nonequilibrium Synthesis and Modulation of Condensed Matter, Xi’an, China, 710049

^3^Harbin Institute of Technology, National Key Laboratory of Science and Technology on Advanced Composites in Special Environments, Harbin, China, 150001

^4^Chinese Academy of Sciences, Shanghai Institute of Optics and Fine Mechanics, Shanghai, China, 201800

^5^Anhui University, State Key Laboratory of Opto-Electronic Information Acquisition and Protection Technology, Hefei, China, 230601

^6^National University of Singapore, Department of Electrical and Computer Engineering, Singapore, Singapore, 117578

*Correspondence:

Longqiu Li, E-mail: longqiuli@hit.edu.cn

Pei Zhang, E-mail: zhangpei@mail.ustc.edu.cn

Gang Gao, E-mail: gaogang@hit.edu.cn

Jiaxin Li, E-mail: [lijiaxin@nus.edu.sg](mailto:lijiaxin@nus.edu.sg)

†These authors contributed equally.

**Note S1. The relationship between the number of eigenmodes of a meta-atom and the independent curvature degrees of freedom of NURBS.**

Near NURBS meta-atoms, the distribution of eigenmodes of the electromagnetic field follows the Helmholtz equation derived from Maxwell's equations^1^,

$\nabla\times\nabla\times E-k^{2}E=0$ (S1)

Below, we use perturbation theory to derive the relationship between curvature and the eigenmode count of NURBS meta-atoms. Assuming that in the absence of perturbation, the eigenvalue of an eigenmode is $k_{0}^{2}$, and the corresponding eigenfunction is $\left\{\psi_{n}^{(0)}\right\}_{n=1}^{\infty}$, which satisfies the orthonormality condition,

$\int_{\Omega} \psi_{m}^{(0)}\left(u,v\right)\cdot\psi_{n}^{(0)}\left(u,v\right)dudv=\delta_{mn}$ (S2)

When curvature (as a perturbation) is introduced, the coefficients of the Helmholtz equation are modified, and the eigenvalues are corrected to $k_{n}^{2}=k_{0}^{2}+\Delta k_{n}^{2}$, where $\Delta k_{n}^{2}$ represents the curvature-induced correction term. According to non-degenerate perturbation theory, the first-order correction is given by,

$\Delta k_{n}^{2}=\left\langle\psi_{n}^{(0)}\left|V\right|\psi_{n}^{(0)}\right\rangle$ (S3)

Here, *V* is the perturbation Hamiltonian, which is directly related to the curvature *K*.

Changes in the curvature *K* alter the metric tensor of the surface, thereby influencing the integral kernel of the Helmholtz equation. Through the pullback mapping of the parameterized surface, the perturbation Hamiltonian can be expressed as,

$V(u,v)=\frac{1}{2}\left(\left|\nabla K\right|^{2}+K^{2}\right)+O$ (S4)

where *O* denotes the higher-order infinitesimal term.

According to the Rayleigh-Ritz principle, the number of modes is determined by the count of modes satisfying $\Delta k_{n}^{2}>\delta$, where$\delta$ represents the spectral interval threshold.

Due to $\Delta k_{n}^{2}\propto\int_{\Omega} \left|\psi_{n}^{(0)}\right|^{2}Vd\Omega$, and $V$ is concentrated in $\Omega$,

$\Delta k_{n}^{2}\approx\int_{\Omega} \left|\psi_{n}^{(0)}(u,v)\right|^{2}V(u,v)dudv$ (S5)

Due to $\psi_{n}^{(0)}$ is localized in high-curvature regions,

$\int_{\Omega} \left|\psi_{n}^{(0)}\right|^{2}\left|K\right|Vdudv\approx\left|\psi_{n}^{(0)}\right|_{\max}^{2}\int_{\Omega} \left|K\right|dudv$ (S6)

Where $\left|\psi_{n}^{(0)}\right|_{\max}^{2}$is the maximum intensity of $\psi_{n}^{(0)}$ within $\Omega$. So we can get,

$\Delta k_{n}^{2}\propto\int_{\Omega} \left|K\right|d\Omega$ (S7)

As $\int_{\Omega} \left|K\right|d\Omega$ increases, the number of modes satisfying $\Delta k_{n}^{2}>\delta$ grows, and consequently,

$N_{m}\propto\int_{\Omega} \left|K\right|d\Omega$ (S8)

And we can get the Eq.1 in the main text.

**Note S2. The geometric characteristics of NURBS and the exposure method of NURBS metasurfaces.**

NURBS (Non-Uniform Rational B-Splines) is a mathematical framework for precise geometric modeling, widely adopted in engineering and design. Its formal definition relies on B-spline basis functions, control vertices, weights, and node vectors, enabling the representation of both analytical shapes (e.g., circles) and free-form surfaces (e.g., turbine blades) with a unified structure.

As shown in Fig. S1a, a B-spline curve of degree *p* is defined by^2^,

$C(u)=\sum_{i=0}^{n} N_{i,p}(u)P_{i}$ (S9)

Where $N_{i,p}(u)$ p-degree B-spline basis function associated with node $u_{i}$, defined recursively as,

$N_{i,0}(u)=\left\{\begin{matrix} \text{1 if }u_{i}\leq u<u_{i+1}\text{ } \\ \text{0 otherwise } \end{matrix}\right.$ (S10)

$N_{i,p}(u)=\frac{u-u_{i}}{u_{i+p}-u_{i}}N_{i,p-1}(u)+\frac{u_{i+p+1}-u}{u_{i+p+1}-u_{i+1}}N_{i+1,p-1}(u)$ (S11)

And $P_{i}$ is control vertex (a point in 2D/3D space), $u$is the parameter in the interval $\left[u_{0},u_{n+1}\right)$. Node vector $U=\left[u_{0},u_{1},...,u_{n+1}\right]$, which dictates the "knotting" (non-uniform spacing) of basic functions.

A rational B-spline curve (a precursor to NURBS) introduces weights $w_{i}$ to enhance shape control,

$R(u)=\frac{\sum_{i=0}^{n} w_{i}N_{i,p}(u)P_{i}}{\sum_{i=0}^{n} w_{i}N_{i,p}(u)}$ (S12)

Such curves can form the sidewalls of a NURBS meta-atoms. A NURBS meta-atom is a modular, parametric unit cell in a metasurface, where geometric properties (e.g., curvature, aspect ratio) are directly controlled by the NURBS formulation above. Such sidewalls can be represented as NURBS surfaces,

$S(u,v)=\frac{\sum_{i=0}^{m} \sum_{j=0}^{n} w_{i,j}N_{i,p}(u)N_{j,q}(v)P_{i,j}}{\sum_{i=0}^{m} \sum_{j=0}^{n} w_{i,j}N_{i,p}(u)N_{j,q}(v)}$ (S13)

The curvature $K\left(u,v\right)$ of the NURBS surface depends on the second derivatives of $S\left(u,v\right)$ with respect to $u$ and $v$. This curvature is not the Gaussian curvature. Fabricating NURBS metasurfaces requires electron beam lithography (EBL) exposure methods to resolve the curvature dependent geometric variations of each super-element. For a given NURBS meta-atom, there exist high curvature regions and low curvature regions. The high curvature regions, with their sharp geometric features (such as edges and corners) and larger curvature values$\left|K\left(u,v\right)\right|$, require lower exposure doses due to their need for high-resolution patterning. In contrast, the low curvature regions, with smaller $\left|K\left(u,v\right)\right|$, characterized by gentle slopes or flat areas, can tolerate higher exposure doses. By leveraging the NURBS mathematical framework, which explicitly encodes curvature through control vertices $P_{i,j}$, weights$w_{i,j}$, and basis functions $N_{i,p}(u)$, NURBS metasurfaces enable precise, curvature-tailored electromagnetic responses ranging from beam steering to adaptive cloaking.

The NURBS meta-atom design framework demonstrates remarkable flexibility in generating diverse geometric configurations and their corresponding electromagnetic responses. As illustrated in Fig. S1a, the NURBS curve is constructed using control points (P0-P4) with associated weights (wᵢ) and a knot vector U, enabling precise parametric control of the meta-atom geometry. Panel (b) presents the fundamental geometric parameters of a 2D NURBS meta-atom, characterized by a degree of freedom (DOF) of 2 × m, with adjustable width and height dimensions centered around point K. Panel (c) showcases six representative NURBS generated meta-atom geometries, including circular, bumped, indented, flower-shaped, square, and oval configurations, along with their eigenmode field distributions at resonant wavelengths ranging from 520 nm to 580 nm. The eigenmode patterns reveal distinct electromagnetic field localization characteristics unique to each geometry, demonstrating the capability of NURBS based parametrization in tailoring optical resonances for meta-surface applications.

**Figure S1.** NURBS Meta-atom Components and Electromagnetic Response Characteristics. (a) Schematic illustration of NURBS curve construction with control points (P0-P4), weights (wᵢ), and knot vector U. (b) Two-dimensional representation of a NURBS meta-atom with degree of freedom (DOF: 2 × m), showing height and width parameters with centroid K. (c) Geometric designs and corresponding eigenmode distributions of six NURBS-based meta-atom configurations. Left panels show the geometric profiles, while right panels display their electric field eigenmode patterns at different wavelengths (λ = 570 nm, 580 nm, 520 nm, 520 nm, 520 nm, and 540 nm, respectively).

In Eq. 2 in the main text, the parameter $c$, which correlates the geometry of a NURBS described meta-atom with the required EBL exposure dose, is implemented using a deep neural network (DNN). This approach is adopted to move beyond empirical fitting and to capture the complex, often non-linear, relationship between the NURBS control point coordinates and the optimal exposure dose observed in experimental fabrication.

The DNN is a fully connected feedforward network trained on a dataset compiled from extensive EBL processing experiments. The input layer consists of nodes corresponding to the coordinates of the NURBS control points defining a meta-atom’s shape. After several hidden layers with non-linear activation functions (e.g., ReLU), the network outputs a single value: the parameter $c$. The dataset includes a diverse set of meta-atom geometries and their corresponding successfully fabricated counterparts, with the target value derived from the measured ideal exposure dose for each geometry (using $c=dose/k$).

The network is trained to minimize the mean squared error between its predicted and the experimental target values. Once trained, the model can predict a geometry optimized for any new NURBS meta-atom design in real time. This data driven method significantly improves pattern fidelity by accounting for subtle, experience based process interdependencies that are difficult to model analytically, thereby enabling more accurate and automated dose assignment for complex metasurface devices like the metalens discussed in the main text.

**Note S3. The derivation of boundary contour sensitivity for NURBS meta-atoms.**

As shown in Fig. S2, the boundary of the meta atoms is $h(s)$. Therefore, the relative permittivity distribution can be expressed as,

$\varepsilon(r)=\varepsilon_{1}+(\varepsilon_{2}-\varepsilon_{1})\Theta(r-h)$ (S14)

where $r$ is the coordinate perpendicular to the boundary direction, and its value at the boundary is 0. And $\Theta$ is the unit step function. The optimization objective of the meta-unit is a function of the phase and/or amplitude of the transmitted optical field. For the electromagnetic system described by Eq. 1, solving the sensitivity of the optical response to geometric boundaries requires the use of the adjoint method. The adjoint method calculates the sensitivity based on the electric field distributions of the original system and the adjoint system. The original system and the adjoint system can be denoted as^3, 4^,

$\nabla\times\nabla\times E-\left(\frac{\omega}{c}\right)^{2}\varepsilon E=J$ (S15)

$\nabla\times\nabla\times E-\left(\frac{\omega}{c}\right)^{2}\varepsilon E=J_{adj}$ (S16)

where $J$ denotes the original excitation source, and $J_{adj}$ represents the adjoint excitation source. Taking the partial derivative with respect to $\varepsilon$ on both sides of Eq. 15 yields,

$\frac{\partial A}{\partial\varepsilon}E+A\frac{\partial E}{\partial\varepsilon}=0$ (S17)

Where $A=\nabla\times\nabla\times-\left(\frac{\omega}{c}\right)^{2}\varepsilon$.Thus, the sensitivity of the optimization objective with respect to the relative permittivity can be derived,

$\frac{\partial F}{\partial\varepsilon}=\frac{\partial F}{\partial E}\cdot\frac{\partial E}{\partial\varepsilon}=\frac{\partial F}{\partial E}\cdot\left(-A^{-1}\frac{\partial A}{\partial\varepsilon}E\right)=-\left(\frac{\partial F}{\partial E}\cdot A^{-1}\right)\frac{\partial A}{\partial\varepsilon}E$ (S18)

Here we set $\lambda^{\dagger}=\frac{\partial F}{\partial E}\cdot A^{-1}$,denotes the conjugate transpose. Thus, by solving follow equation, the corresponding $\lambda^{\dagger}$ can be obtained,

$A^{\dagger}\lambda^{\dagger}=\left(\frac{\partial F}{\partial E}\right)$ (S19)

Thus, in Eq. 15,

$J_{adj}=\frac{\partial F}{\partial E}$ (S20)

The solution of the adjoint system, $E_{adj}$, can be obtained by solving either Eq. 15 or Eq. 18. Substituting into Equation 17 yields the sensitivity as,

$S=\frac{\partial F}{\partial\varepsilon}=-\left(\frac{\partial F}{\partial E}\cdot A^{-1}\right)E\frac{\partial A}{\partial\varepsilon}=-E\cdot E_{adj}\frac{\partial A}{\partial\varepsilon}$ (S21)

In NURBS meta-atoms, variations in geometric parameters only cause boundary displacement. Based on Eq. 14, boundary perturbation can be expressed as,

$\frac{\delta\varepsilon}{\delta h}=(\varepsilon_{2}-\varepsilon_{1})\delta(r-h)$ (S22)

Where $\delta(r-h)$ is Dirac function. Thus, the contribution of perturbation on the boundary of the NURBS meta-atoms is,

$\frac{\partial F}{\partial s}=(\varepsilon_{2}-\varepsilon_{1})\int\left(\frac{\partial A}{\partial\varepsilon}E_{adj}E\right)\frac{dh}{ds}\delta(r-h)dS$ (S23)

Here $s$ is the design parameter. However, since the electric field in a continuous medium exhibits continuous normal components and discontinuous tangential components at the interface between two dielectric materials, the relative permittivity on the sidewall boundary of the NURBS super-element cannot be treated as a uniform scalar value^1^. Instead, it must be equivalently represented as a second-order tensor $\left(\begin{matrix} \varepsilon_{\perp} & & \\ & \varepsilon_{\parallel} & \\ & & \varepsilon_{\parallel} \end{matrix}\right)$. This implies that both the electric field and the adjoint field need to be computed separately for their tangential and normal components.

$\frac{\partial A}{\partial\varepsilon}E_{adj}E=\Delta\varepsilon_{12}E_{\parallel adj}E_{\parallel}-\Delta(\varepsilon_{12}^{-1})D_{\perp adj}D_{\perp}$ (S24)

Where $\Delta\varepsilon_{12}=\varepsilon_{2}-\varepsilon_{1}$ and $\Delta(\varepsilon_{12}^{-1})=\frac{1}{\varepsilon_{2}}-\frac{1}{\varepsilon_{1}}$. $D$ represents the electric displacement (field). Finally, we obtain,

$\frac{\partial F}{\partial s}=-\left(\frac{\omega}{c}\right)^{2}\int\frac{dh}{ds}[\Delta\varepsilon_{12}E_{\parallel adj}E_{\parallel}-\Delta(\varepsilon_{12}^{-1})D_{\perp adj}D_{\perp}]dS$ (S25)

This equation quantifies the sensitivity of the meta-atom's performance to variations in its boundary geometry. It serves as a critical guiding principle for performing inverse design and structural optimization of the meta atoms. After replacing full-wave simulations with a surrogate model, the automatic differentiation property of deep learning enables immediate extraction of the corresponding sensitivity or gradient of the current structure. In practical engineering applications, the derived process described above can be employed to validate the accuracy of the surrogate model. When sufficient computational resources are available, the sensitivity information obtained from the aforementioned analysis can be incorporated as training data to further train the surrogate model. This enhances the model's alignment with the physical structural properties.

**Figure S2.** Perturbing the NURBS meta-atoms. The structure of a NURBS meta atoms can be composed of two materials with different relative permittivity. When a perturbation is applied at the boundary, it is primarily the electric field distribution near the boundary that dictates the overall unit's sensitivity.

**Note S4.** Training convergence and validation of the Transformer surrogate model.

Figure S3 provides an additional validation of the Transformer surrogate model used for NURBS meta-atom optical-response prediction. The plotted curves show the first 500 epochs extracted from the full 10,000-epoch training process, which capture the rapid early-stage convergence of both the training and validation losses. The validation metrics further evaluate the predicted complex response, amplitude, and phase, confirming that the surrogate reaches a stable prediction regime before the selected best epoch. This supplementary analysis supports the use of the trained surrogate model for rapid wavelength-dependent phase and transmittance prediction in the inverse-design workflow.

**Figure S3.** Training convergence and validation performance of the Transformer surrogate model. The plotted curves show the first 500 epochs extracted from the full 10,000-epoch training process. The training and validation losses decrease rapidly during the early stage and then gradually converge. The validation metrics, including complex MAE, amplitude MAE, and phase MAE, indicate stable surrogate prediction performance, with the best validation epoch identified at epoch 385.

**Note S5.** The preparation method of mouse pancreatic cancer cells.

As shown in Fig. S4, the mouse pancreatic cancer cells (Panc02) were purchased from Wuhan Procell Life Science & Technology Co., Ltd. and then cultured with DMEM [Procell, PM150210] containing 10% FBS [Procell, 164210] and 1% P/S [Procell, PB180120] in cell-cultured incubator at 37 °C with 5% CO2. For preparation of cell coverslips, 5×103 cells were seeded on 24×24 mm sterile glass coverslips in 6-well plates. Cultures are maintained until reaching 50-80% confluency. Then coverslips were washed twice with PBS and fixed with 4% paraformaldehyde.

**Figure S4.** Workflow for Panc02 cell culture and fixation on glass coverslips. Panc02 cells were cultured in DMEM supplemented with 10% FBS and 1% P/S at 37°C in a 5% CO2 humidified incubator until reaching 50-80% confluency. For immunofluorescence experiments, cells were seeded onto 24×24 mm glass coverslips at a density of 5×10⁴ cells per coverslip, washed twice with PBS, and fixed with 4% paraformaldehyde.

**Note S6. Radial contour-deviation analysis of fabricated NURBS meta-atoms.**

To provide a quantitative structural comparison between the fabricated and designed NURBS meta-atoms, representative unit cells were selected at different radial positions across the 200-μm-diameter metalens. For each selected position, the SEM-extracted boundary was registered to the corresponding designed NURBS contour, and the pointwise Euclidean distance between the two contours was integrated along the boundary. The integrated L2 contour deviation was defined as:

$D_{int}=\int_{0}^{L} \left\|r_{SEM}\left(s\right)\text{ }-\text{ }r_{design}\left(s\right)\right\|_{2}ds$ (S26)

where$r_{SEM}\left(s\right)$ and $r_{design}\left(s\right)$ represent the SEM-extracted and designed contour coordinates parameterized by the boundary arc length *s*, and *L* is the total contour length. Because the pointwise distance and the integration variable both have length units, Dint has units of nm². This metric provides a compact measure of the accumulated boundary mismatch between the fabricated and target NURBS profiles. The radial distribution shown in Fig. S5 indicates that the contour deviation remains at a comparable level over the sampled radial positions, supporting consistent transfer of the main NURBS freeform boundary features across the metalens aperture.

**Figure S5.** Radial contour-deviation analysis of fabricated NURBS meta-atoms across the 200-μm-diameter metalens. Representative NURBS meta-atoms were selected at different radial positions from the lens center, and their SEM images were compared with the corresponding designed NURBS profiles. The lower panel shows the integrated L2 contour deviation as a function of radial position, calculated by integrating the pointwise Euclidean distance between the SEM-extracted contour and the designed NURBS contour along the boundary. Error bars indicate the uncertainty associated with contour extraction and image registration. The comparable deviation values over the sampled radial positions provide structural evidence that the main NURBS boundary features are consistently transferred across the metalens aperture.

**References**

1. S. G. Johnson, M. Ibanescu, M. A. Skorobogatiy, et al. “Perturbation theory for Maxwell's equations with shifting material boundaries,” *Physical Review E* 65, no. 6 (2002): 066611, https://doi.org/10.1103/PhysRevE.65.066611
2. L. Piegl and W. Tiller, *The NURBS book* Springer Science & Business Media (2012).
3. T. W. Hughes, M. Minkov, I. A. D. Williamson and S. Fan. “Adjoint Method and Inverse Design for Nonlinear Nanophotonic Devices,” *ACS Photonics* 5, no. 12 (2018): 4781-4787, https://doi.org/10.1021/acsphotonics.8b01522
4. M. Minkov, I. A. D. Williamson, L. C. Andreani, et al. “Inverse Design of Photonic Crystals through Automatic Differentiation,” *ACS Photonics* 7, no. 7 (2020): 1729-1741, https://doi.org/10.1021/acsphotonics.0c00327
